# Supplementary material for: Prospective estimation of the age of initiation of cigarettes among young adults (18–24 years old): Findings from the Population Assessment of Tobacco and Health (PATH) waves 1–4 (2013–2017)
Source: PLoS One. 2021 May 5;16(5):e0251246. doi: 10.1371/journal.pone.0251246 (PMC8099124; doi:10.1371/journal.pone.0251246)
Supplement: S1 Table — (DOCX) [file pone.0251246.s001.docx]

**Pérez. A, Kuk, A.E., Bluestein, M.A., Penedo, E., N’hpang, R.S., Chen, B., Perry, C.L., Sterling, K.L., Harrell, M.B**. **(2021) Prospective estimation of the age of initiation of cigarettes among young adults (18-24 years old): findings from the Population Assessment of Tobacco and Health (PATH) waves 1-4 (2013-2017). PLOS ONE.**

**Supplemental Table 1: Total number of other tobacco products, excluding e-cigarettes, ever used prior to the initiation of cigarette use outcomes among PATH young adult (ages 18-24 years old) never cigarette users at their first wave of adult study participation, 2013-2016.**

| **Never cigarette users at first wave of adult study participation** | | | |
| --- | --- | --- | --- |
|  | | n=5,523; N=19,548,811^a^ | |
|  |  | Unweighted n (N) | Weighted % (SE) ^b^ |
| Total number of other tobacco products ever used, except e-cigarettes, prior to ever cigarette use* | 0 | 3,594 (13,520,564) | 69.2 (1.02) |
|  | 1 | 1,050 (3,260,432) | 16.7 (0.69) |
|  | 2 | 400 (1,172,802) | 6.0 (0.36) |
|  | 3 | 197 (542,134) | 2.8 (0.27) |
|  | 4 | 77 (214,140) | 1.1 (0.14) |
|  | 5 | 26 (73,379) | 0.4 (0.08) |
|  | Missing | 179 (765,360) | 3.9 (0.43) |
| Total number of other tobacco products ever used, except e-cigarettes, prior to past 30-day cigarette use* | 0 | 3,566 (13,372,706) | 68.4 (1.04) |
|  | 1 | 1,049 (3,285,140) | 16.8 (0.71) |
|  | 2 | 413 (1,234,441) | 6.3 (0.37) |
|  | 3 | 208 (584,982) | 3.0 (0.28) |
|  | 4 | 78 (214,379) | 1.1 (0.14) |
|  | 5 | 30 (86,065) | 0.4 (0.08) |
|  | Missing | 179 (771,099) | 3.9 (0.43) |
| Total number of other tobacco products ever used, except e-cigarettes, prior to fairly regular cigarette use* | 0 | 3,515 (13,168,088) | 67.4 (1.04) |
|  | 1 | 1,051 (3,339,542) | 17.1 (0.71) |
|  | 2 | 425 (1,275,863) | 6.5 (0.38) |
|  | 3 | 231 (657,272) | 3.4 (0.30) |
|  | 4 | 91 (250,059) | 1.3 (0.16) |
|  | 5 | 36 (106,472) | 0.5 (0.09) |
|  | Missing | 174 (751,516) | 3.8 (0.43) |

* PATH restricted file received disclosure to publish: March 09, 2021. United States Department of Health and Human Services. National Institutes of Health. National Institute on Drug Abuse, and United States Department of Health and Human Services. Food and Drug Administration. Center for Tobacco Products. Population Assessment of Tobacco and Health (PATH) Study [United States] Restricted-Use Files. ICPSR36231-v13.AnnArbor, MI: Inter-university Consortium for Political and Social Research [distributor], November 5, 2019. <https://doi.org/10.3886/ICPSR36231.v13>.

a: n= unweighted frequency; N= weighted frequency

b: SE= standard errors
