# Supplementary material for: Prospective estimation of the age of initiation of cigarettes among young adults (18–24 years old): Findings from the Population Assessment of Tobacco and Health (PATH) waves 1–4 (2013–2017)
Source: PLoS One. 2021 May 5;16(5):e0251246. doi: 10.1371/journal.pone.0251246 (PMC8099124; doi:10.1371/journal.pone.0251246)
Supplement: Attachment — Submitted filename: ResponsetoReviewers_PONED2026733_03_17_2021.docx [file pone.0251246.s002.docx]

This is our rebuttal letter that responds to each point raised by the reviewers and the academic editor. We used a table description to facilitate this reading. The lines stated are referring to the track changes version to facilitate its identification on the paper. Thank you for reading.

| **Academic Editor** | **Answers from Authors** |
| --- | --- |
| Please ensure that your manuscript meets PLOS ONE's style requirements, including those for file naming. | We have reviewed and our manuscript meets PLOS ONE’ style requirements. Thank you for your feedback in helping us to improve this paper. |
| In your statistical analyses, please state whether you accounted for repeated measurements and the potential effect of clustering by state. For example, did you consider using multilevel models? | Our analysis of the PATH dataset is using SURVIVAL analyses to estimate the age of initiation of cigarettes in young adults. Multilevel models are not appropriate for the outcome of interest: the age of initiation of cigarettes in young adults. Multilevel models are appropriate if the event is binary or categorical across time. This is not our case. We do not have the issue of accounting for the repeated measures of cigarette use in survival analysis as once the never users of cigarette report the use they initiated at whatever wave. Per our training in the use of the PATH dataset the clustering effects by the Primary sampling units (PSU) are not necessarily by state as the PSUs does not necessarily mimic the states in the USA. For sure we account for the complex sampling design and the multiple waves of PATH data by using the sampling weights of each participant at the first wave of entry into PATH. |
| Reviewer 1: Is the manuscript technically sound, and do the data support the conclusions? | We respectfully disagree with Reviewer 1, as we feel that our manuscript is technically sound. |
|  |  |
| **Reviewer #1** | **Answers from Authors** |
| The manuscript entitled “Prospective estimation of the age of initiation of cigarettes among young adults (18-24 years old): findings from the Population Assessment of Tobacco and Health (PATH) waves 1-4 (2013-2017)” addresses the important topic of late initiation of cigarette smoking, at ages 18-24 which is past the ages when the vast majority of smokers begin smoking. Thus, this broad topic has potentially important public health implications. | Thank you for your feedback in helping us to improve this paper. |
| A great strength is that this research is embedded within the PATH Study, a very rich nationally representative resource with longitudinal data to assess this question. Further, the research team seemed to have excellent expertise in the data management and statistical analytic approaches to maximize the use of these detailed data. An interesting finding was that whereas individuals who identify as non-Hispanic White are much more likely to start smoking at younger ages compared with other racial/ethnic groups, this seems to be reversed during the 18-24 year-old age period. | Thank you for your feedback in helping us to improve this paper. |
| 1. It was a negative beginning to have an abstract with no scientific rationale or background to provide context, creating the impression from the outset that the quantitative expertise is not balanced by substantive expertise. | The scientific rationale behind our study is that there have been increases in recent years in the proportion of cigarette initiation occurring in young adulthood^1^. We have added this background for context in the abstract: (lines 26-27)  “**given recent increases in cigarette initiation occurring in young adulthood.”**  In addition, we have added this into our introduction providing stronger context for the need of the results of our manuscript in Lines 68-71 of the tracked version:  **“A recent study of the cross-sectional NSDUH 2002-2018 found that the recalled age of cigarette initiation occurring in young adulthood (18-23 years old) increased from 20.6% in 2002 to 42.6% in 2018, indicating that young adults are increasingly vulnerable to initiating cigarette use”.** |
| 2. The very first sentence of the manuscript needs work. With cigarette smoking, providing a partial list of smoking-caused diseases is not informative enough and referring to cigarette smoking as “…one of the associated factors of…death in the United States” grossly understates its importance as the leading cause of premature death estimated to cause 480,000 deaths per year. Further, cigarette smoking has not been found to be a cause of breast cancer. | Thank you for pointing this out. We agree that stronger language on the relationship between cigarette use and adverse health outcomes will enhance the manuscript. Lines 55-57 have been revised:  **“cigarette use remains a leading cause of one of the associated factors of many preventable diseases (i.e., cardiovascular disease, emphysema, chronic bronchitis, breast cancer, and lung cancer, among others), and death in the United States”** |
| 3. In the Introduction, in the first sentence of the second paragraph a seemingly identical publication using PATH Study data (but with shorter follow-up) is referenced that includes members of the same research team as this manuscript. This reduced enthusiasm about the novelty of this research, making it seem like little advance over the already published work (reference 9). | There are several differences between our previous publication^2^ and the current study besides the follow-up period. In the previous publications we reported proportions of initiation in two age groups: 12-17 year olds and 18-24 year olds, while the current study provided the hazard function of age of initiation. Another difference between the 18-24 year olds in Perry et al.^2^ and this paper is that in the previous paper we only included 18-24 year olds at wave 1, while in this manuscript we include 18-24 year olds at wave 1 and youth who turn 18 at wave 2 or wave 3. Thus, our follow-up period for this paper is much longer. The reviewer is correct that Perry et al.^2^ had 1 year of follow-up, while the current study has up to 4 years of follow-up. Third, we include fairly regular use as an outcome while Perry et al.^2^ does not. Finally, but most importantly, we use interval-censored weighted survival analysis, which uses initiation + age instead of just proportions within an entire age group. This is documented in the methods section. |
| 4. In the current complex tobacco marketplace precision in identifying products assumes increased importance. It is helpful to define terms early on, to make clear to the reader that when the manuscript refers to “cigarettes” this is referring to “combustible tobacco cigarettes” (or something of the sort). | Thank you for pointing this out. We have added the words “combustible tobacco cigarettes” to the manuscript to clarify for our readers. Line 54. |
| 5. It was puzzling that the manuscript seemed to be focused on 18-24 year-olds from the title onward, but then all of the sudden data is presented about 27 and 28 year-olds. Presumably, this is due to the 23-24 year-olds at baseline being followed over time, but this age issue needs to be clarified early on and handled uniformly throughout the manuscript. | Yes, the reviewer is understanding correctly, there are young adults who were 24 years old at wave 1 therefore by wave 4 their age could have been 28 years old. For example, if a participant was 24 years old and 51 weeks, by wave 4 they would be 28+ years old. Lines 118-136 describe the data management and follow-up periods for participants and Figure 1 depicting this has been added for clarity. We have added lines 134-136 to clarify the follow-up time of our longitudinal study and participant ages to our readers.  We have also clarified in lines 183-185 that the follow-up time between waves could have exceeded 52-53 weeks, but participants were measured once per year. |
| 6. Following up on Comment #5, the biggest increase occurring between 18 and 19 years-old is completely expected and makes sense, but how could the second biggest increase in past 30-day use be among 27 to 28 years-old? This does not adhere to prior expectation and thus deserves more attention. | We want to thank the reviewer for their meticulous review of the results. We have some unfortunate news. We regret that after taking a closer look at the results in the original output, we realize that the confidence intervals were very wide. We also realized that our Ms N’Hpang did not transcribe the values correctly. Thus, there were multiple errors in Table 2. Table 2 has now been fixed and we confirm that the values for age 28 were incorrect because the output showed that there was not enough sample size to estimate initiation at that age. Given the problems named above, we double-checked all the results in all the tables, and found similar issues with Table 4 and the results for ever use, as they were not transcribed correctly either. Thus, we recreated Table 4 to reflect the correct results. No errors were found in Table 1 or Table 3 from our previous submission. Tracked changes were not used for Table 2 and Table 4 because of the multiple changes that had to be made. Their content has been reproduced entirely.  Despite the changes that had to be made to Table 2 and Table 4, fortunately, this did not change any of the conclusions that were originally made from this manuscript. We confirmed that all figures are correct because these figures use the full distribution of ages instead of extracting results from the output. |
| 7. In Table 2 the terminology “hazard functions” is used over the more accessible and public health relevant term “cumulative incidence” (which is relegated to a footnote). Changing this will improve clarity for readers from different disciplines. | We have changed the terminology from “hazard functions” to “cumulative incidence” for both tables 2 and 4, as well as Figures 2-4. |
| 8. An extremely important variable to consider in this research is prior tobacco product use. The authors accounted for this by creating a variable that summed up the number of products used in the past (0, 1, and >=2). While this has relevance, a question of burning scientific interest is the relationship between prior use of a specific tobacco product and the risk of future smoking—that specific tobacco product is e-cigarettes. So, this was a major missed opportunity to document the association between prior e-cigarette use in relation to future initiation of cigarette smoking in young adulthood. Even so, the importance of prior tobacco product use is clear by the very strong associations between prior tobacco product use and future cigarette smoking. A p-value for trend should be included for this variable (likely highly statistically significant in every model). Remarkably, this strong association was not mentioned in the Discussion section when it should have been a major focus. Overall, the handling of other tobacco product use was a missed opportunity and this was rated as a major weakness. | Thank you for pointing this out. We want to clarify the focus of this paper was not about previous e-cigarette use and its association with subsequent cigarette use, which is a different research question in our humble opinion. However, to comply with this reviewer request, we have updated our analysis, methods, results, and discussion sections to reflect the importance of this association. We agree with the reviewer about the importance of controlling for previous other tobacco product use and the priority to separate previous e-cigarette use from previous use of other tobacco products. Therefore, we have adjusted for these as separate variables in analyses.  We created three new variables that reflect ever e-cigarette use prior to ever, past 30-day, and fairly regular cigarette initiation. We have also created 3 “other tobacco product use” variables to reflect ever use of 5 tobacco products (traditional cigar, cigarillos, filtered cigar, hookah, smokeless tobacco) except cigarettes and e-cigarettes so that they are independent from each other that are created to occur before each of the 3 cigarette use outcomes. Lines 150-164 reflect these changes. We report these new results in Table 3, we updated the methods section to describe these 2 new variables and have added the interpretation in the Results and Discussion sections to reflect this update. We did not use the track changes feature for Table 3 due to so many changes.  We don’t report a p-value for a trend analysis because we are not using the proportions to estimate the longitudinal association across waves. Instead, we are estimating the effect of prior e-cigarette/other tobacco product use on the age of initiation of cigarettes using survival analysis, where trend analysis does not apply as the time to event is estimated in the outcome instead of consider it as an exposure. |
| 9. In the Discussion section, the text in lines 363 to 367 attempts to delve into disparities in lung cancer which the authors eventually state is beyond the scope of this research—this text should be omitted from the manuscript for this very reason. | We have removed these lines from the text. |
| 10. The manuscript ended abruptly with “Strengths and Limitations” without a synopsis of the findings and conclusions about the results. | We have added a conclusion section to provide an explicit conclusion to the manuscript Lines 530-538:  “**In conclusion, this study provides strong evidence that a substantial portion of contemporary cigarette initiation does occur in young adulthood and gives estimates for the age of cigarette initiation. Among 18 year olds who have never smoked a cigarette, 22.7% report initiating conventional cigarette smoking by the age of 27. In addition, males, Hispanic young adults, Non-Hispanic Black young adults, previous e-cigarette users and those who have used other tobacco products before cigarette initiation have an increased risk of initiating cigarette use at earlier ages. Based on these findings, cigarette prevention and education campaigns should be expanded to target young adults, especially 18-21 year olds.”** |
|  |  |
| **Reviewer 2 Minor Comments** | **Answers from Authors** |
| 1. In the Methods section, line 115-117 there is a statement “…this resulted in 5,523 (N=19,548,811) …” It was unclear what “N” was when this appeared. It becomes clearer later that this “N” stands for the weighted frequency, but this should be clarified when it is first introduced. | We have added the words “weighted frequency” to describe what “N” means. (Line 132) |
| 2. Please mention in Table 1 that the capital N is the weighted frequency in the row immediately below the row titled “Never cigarette users at first wave of adult study participation”. | We have added a footnote to describe that N= weighted frequency. (Line 247) |
| 3. It is very unusual in my peer review experience to have tables embedded directly in the text, and this formatting makes it difficult to read and review the manuscript especially when the tables were broken up across multiple pages. | The formatting requirements for PLOS One include embedding the tables in the manuscript. |
| 4. In lines 181-184 the reader is provided with the information that “Each hazard function per outcome took approximately 30 hours to complete using the restricted-use data server [23], with an additional 30 hours for sex and 30 hours for race/ethnicity, resulting in a total run time of approximately 360 hours (=90 hours*4 outcomes) to complete all analyses.” This sentence is not necessary, and the manuscript would be strengthened by omitting it. | We wanted to share with our readers that if they decide to do a survival analysis with PATH, using the sampling weights means that completion of the analysis will be time-consuming. However, given the reviewers’ request we have removed these sentences. |
| **Reviewer #2:** | **Answers from Authors** |
| This manuscript presents the findings from an analysis of longitudinal PATH data to assess the age of cigarette initiation among young adults aged 18-24 who were never cigarette smokers at baseline. The study found that among young adults who never smoked at baseline, the greatest increase in initiation occurred between 18-19 years old, and by age 21, approximately 1 in 10 had initiated ever cigarette smoking and about 1 in 13 had initiated past 30 day smoking. By demographics, initiation was more likely among males, Hispanics, and non-Hispanic Blacks. Overall, this study is methodologically sound, and would have important implications for informing tobacco prevention strategies among young adults, including regulatory actions by the US Food and Drug Administration. Nonetheless, it could still be improved with some relatively straightforward revisions described below. | Thank you for your feedback in helping us to improve this paper. |
| 1. Page 2. Abstract. Conclusion, First sentence. The primary call to action could be stated more explicitly (e.g. “The prominent initiation of cigarette smoking among young adults reinforces the need for prevention strategies among this population”). | We appreciate this feedback. We agree and we have incorporated it into the first sentence of the conclusion in the abstract. (Lines 45-46) This is the text added:  **“Conclusions: The substantial amount of cigarette initiation among young adults reinforces the need for prevention strategies among this population”.** |
| 2. Page 4. Introduction. First paragraph. Cigarette smoking has only been declining since the mid-1960s among adults. Youth smoking rates increased prominently until the 1998 Master Settlement Agreement, and have been declining since. | We have added the words “among adults” to this finding to be clearer. (Line 55) This is how it reads now:  **“Though the prevalence of combustible tobacco cigarette use has declined since the mid-1960s among adults, cigarette use remains a leading cause of many preventable diseases (i.e., cardiovascular disease, emphysema, chronic bronchitis, and lung cancer, among others) and death in the United States”** |
| 3. Page 5. Introduction. Last Paragraph. It would be helpful if the authors explicitly articulated the inherent novelty of the present study and how it builds about the existing scientific literature. There are references to previous studies, but the Introduction section doesn’t make a clear connection as to how the study specifically fills an existing void. It’s also important that the authors cite several recent PATH studies published on this issue, which aren’t otherwise included. For example, earlier this year Tobacco Control published a special supplement using longitudinal analyses from PATH during 2013-2016, including Stanton et al (initiation among youth, young adults, and adults), and Taylor et al (exclusive and polytobacco cigarette use among youth, young adults, and adults). | We appreciate the reviewer’s knowledge of the PATH literature. We also want to reiterate that none of the PATH studies address the age of cigarette initiation in young adults, which is the focus of our paper. We have added lines 94-96 to clearly state that none of the previous PATH studies prospectively estimated the age of cigarette initiation, which is the goal of our study. While we do make comparisons with the Stanton paper in the discussion, we have also added some commentary on the Stanton paper in the new version of the introduction (Lines 80-82). This is:  **“In addition, a previous report of PATH among young adults reported initiation of ever (10.3%) and past 30-day (6.1%) cigarette use after 1 or 2 years of follow-up”.**  We also want to thank the reviewer for pointing out the importance of the Taylor3 paper, which we originally missed. While the focus of the Taylor3 paper is on longitudinal pathways of exclusive and polytobacco cigarette use, there are some interesting findings from this paper that we have added to the discussion (Lines 470-474). This is the added text:  **“A recent PATH study found that past 30-day poly-tobacco cigarette use (use of cigarettes and at least 1 other tobacco product in the past 30-days) is common among young adults (65.2% of tobacco users) at wave 1, and continued poly-tobacco cigarette use was the most persistent pattern of tobacco across waves 2 and 3 (37.1%).”** |
| 4. Page 6. Study Design and Participants. Given that many readers will not have a nuanced understanding of PATH methods, it would be beneficial if the authors better clarified when waves occurred – e.g. Wave 1 (2013-2014), Wave 2 (2014-2015), etc. It’s also recommended that they use consistent terminology when referencing these periods. For example, in some areas of the manuscript “Wave X” is used, while elsewhere the years of data collection are used, to reference the findings for that period. | We have modified the sentences to include the time period for each wave (Lines 120-122). We have also reviewed the rest of the text and have made changes to refer to all time periods as waves instead of years for consistency. The text is now:  **This study conducted secondary analyses using the PATH restricted-use adult datasets among young adult (18-24 years old) never users of cigarettes in waves 1-3 (wave 1: 2013- 2014, wave 2: 2014-2015, wave 3: 2015-2016) with outcomes followed-up in waves 2-4 (wave 2: 2014-2015, wave 3: 2015-2016, wave 4: 2016-2017).** |
| 5. Page 6. Study Design and Participants. The breakdown of study participants across waves, including excluded participants, is a bit difficult to follow in narrative form. It’s recommended that the authors add a figure with a flow chart that visually clarifies how the final sample was ultimately derived. | We have created a flow chart (Figure 1) to better exemplify the data management and eligibility of participants. |
| 6. Page 7. Cigarette Use Measures. It’s not clear what is meant by “derived variable” for the ever use measure. It’s recommended that more clarity be provided on the precise framing of the question(s) that were used to create this measure. | The derived variable was provided by PATH in the restricted dataset. We did not derive a variable for ever use. We have included the exact wording of the variable for clarity and removed the wording of “derived variable” to avoid confusion that we created this variable ourselves (Lines 140-141).  **“The PATH study at waves 2-4 asks participants: “Have you ever smoked a cigarette, even one or two puffs?”.** |
| 7. Page 7. Other Tobacco Product Use. It’s not clear why the authors only examined ever tobacco product use at participants’ first PATH wave of participation. Couldn’t initiation have also occurred during a subsequent wave (e.g. wave 2), prior to assessment over time (e.g. wave 4)? Further clarity on this analytic choice is warranted to better justify the approach to readers. | We agree with the reviewer that a superior measure of “other tobacco use” would reflect any tobacco use prior to cigarette initiation and not just at the participants’ first wave of PATH participation. We created three new variables to represent any tobacco use prior to ever, past 30-day, and fairly regular cigarette initiation (Lines 150-164 methods). We report these results in Table 3 and have modified the Results and Discussion sections to reflect this update. Please see our response to reviewer 1 comment 8 for more details. |
| 8. Page 10. Table 1. The weighted percentages in the table are confusing, particularly having missing values in excess of 8,000 for the sex category. It would be much more straightforward for the reader if the authors included unweighted sample sizes in this table (or both unweighted and weighted if the authors feel strongly). | At the time of writing this manuscript, ICPSR which houses the PATH data did not allow the release of results with unweighted frequencies from their restricted-use server. However, their policy has changed, and they now allow us to publish results with unweighted frequencies, so we have added the unweighted frequencies to Table 1. We have kept the weighted frequencies clarifying that they represent the population estimates to the readers in both table and text. |
| 9. Page 17. Discussion. First paragraph. The authors reference a “past 30 day” estimate from the National Health Interview Survey (NHIS), but current use from that survey is usually reported using a 100 lifetime threshold and “everyday”/”someday” use. Please cross-check the actual reference to ensure that the language you’ve used is accurate as framed. | We want to thank the reviewer for pointing out this discrepancy. We have updated the manuscript to reflect this difference and to differentiate their results from ours we use “current” use (lines 391-396).  **“While the results from the 2018 National Health Interview Survey (NHIS) suggested that an estimated 13.7% (34.1 million) of US adults are current cigarette users (has smoked more than 100 cigarettes in their lifetime and smoked every day or some days at the time of the survey) and 7.8% of young adults (18-24) were current users of cigarettes, our study estimated more than 3.13 million (19,548,811*0.160) past 30-day cigarette users initiate by the age of 27 between 2013-2017.”** |
| 10. Pages 17-18. Discussion. The authors nicely describe their findings in comparison to other studies, including the variations by sex and race/ethnicity. However, the utility and relevance of this manuscript would be greatly enhanced if the authors explained why these disparities are likely occurring, including potential drivers that are creating these inequities. | We agree that an explanation for the sex difference in the age of cigarette initiation would be enhance the paper. However, while there is a plethora of research on sex differences in the proportion of cigarette use, there is a lack of research on sex differences in the age of cigarette initiation, which is a few finding from this paper. In addition, research on sex differences in the age of cigarette initiation have largely been conducted in youth, and the risk factors that predispose male youth to an earlier age of cigarette initiation may be different for male young adults. We have added this as a direction for future research on lines 409-413.  **“Most of the research on sex differences in the age of cigarette initiation have been conducted among youth, so more research is needed to determine the risk factors that predispose males to an earlier age of cigarette initiation in young adulthood, as the risk factors that predispose male youth to an earlier age of cigarette initiation may be different for young adult males“**  We agree that exploring protective and risk factors that could explain the race/ethnicity disparities are of interest, however, there is a lot of research exploring those correlates with the initiation of cigarettes. In this paper, we did not consider exploring correlates for the age of initiation of cigarette use among young adults because we did not know if there were going to be differences by race/ethnicity and with a correlation study we would limit our sample size even further by including more risk factors to those who had complete data for those variables. However, we have included possible explanation for race/ethnicity differences that have been studied in other research on lines 434-450.  **“The increased risk for initiating cigarette use among Non-Hispanic Black young adults could be explained by Minority Diminished Return theory, which posits that the factors known to be protective against cigarette use in Non-Hispanic Whites are less protective among Non-Hispanic Blacks. Observed racial inequalities in the education system, labor market, and other institutions contribute to these diminished protective effects. For example, the educational system has been shown to discriminate against African Americans, reducing the gain that comes with higher education. The labor market discriminates against African Americans in hiring and wages, reducing the protection that comes with full-time work. These explanations are extremely important to consider in future research in order to combat the health effects associated with institutionalized racism. In terms of the current study, an earlier age of cigarette initiation among Non-Hispanic Black young adults increases the number of years of cigarette use, which increases the risk of adverse health consequences. Other possible explanations include predatory marketing by the tobacco industry in low socioeconomic status (SES) and high minority neighborhoods, flavor branding, SES, quality of healthcare, among others. The current study findings highlight a need for future studies to examine potential risk and protective drivers of tobacco use disparities exist across these groups.”** |
| 11. Page 18. Discussion. The authors appropriately call for prevention interventions among young adults, but it could be more clearly stated in the narrative (see comment #1 above). Additionally, it would be immeasurably more helpful if the authors explained what interventions they’re talking about. The most effective prevention measures are population-based policies, of which they are many, including price, smoke-free, tobacco 21, and even product restrictions (e.g. flavors). At present, there’s a lot of talk about FDA regulation, which is fine – but states and communities are not preempted by the Family Smoking Prevention and Tobacco Control Act, and indeed much of the momentum on population-based policies are presently occurring at the state and local level. Not acknowledging any of the strategies that can move the needle forward on preventing young adult use at the national, state, and local levels is really a missed opportunity here. | We appreciate the reviewer turning our attention to population-based strategies for prevention interventions. We have reviewed the literature and included references to emphasize the importance of these strategies for reducing cigarette initiation (lines 492- 500).  **“In addition, population-based strategies, such as FDA’s required cigarette health warnings that include text, color graphics, and descriptions of some of the less-known but serious health risks from cigarette use, will be paramount to reduce cigarette initiation overall. Other effective strategies that can be implemented at the population level include mass media campaigns, comprehensive smoke-free policies, state-funded cessation programs, reducing the nicotine content in cigarettes, menthol bans, and increasing the tax on cigarettes. Additional research is needed to better inform the development of culturally-relevant tobacco prevention interventions for young adults, and to evaluate the reach and coverage of those interventions.”** |
| 12. Page 19. Discussion. The authors call for culturally relevant prevention interventions, which is reasonable. But they also need to reinforce that equitable coverage of interventions is needed in the first place. Ultimately, being covered by an intervention in the first place is going to be a more critical line of defense when it comes to prevention than a targeted intervention. | We appreciate the reviewer turning our attention to population-based strategies for prevention interventions. We have reviewed the literature and included references to emphasize the importance of these strategies for reducing cigarette initiation. Text was stated before in reviewer 1 comment 11. |
| 13. Page 19. The authors reference the “Tobacco 21 Law”, but this won’t be intuitive to lay readers. There needs to be more context around this statement, which presumably references the Federal law adopted in December 2019 to increase the age of sale nationally to 21.  That said, it would also seem relevant to reference the numerous state and local tobacco 21 laws that were also implemented prior to the national law.  Also, it’s not clear why the authors have just focused on advertising and marketing at the end of this paragraph. There are numerous regulatory actions related to manufacturing, marketing, and sale that could be taken by FDA to mitigate young adult initiation besides just addressing advertising and marketing (e.g. menthol, nicotine reduction, etc). | We have added a description of the Tobacco 21 law to enhance clarity for our readers (lines 501- 504).  **“Finally, our study can be used as a baseline for any future evaluation of the effectiveness of the Tobacco 21 law, a U.S. federal law that changed the minimum age of tobacco sale from 18 years old to 21 years old in December 2020, to determine if this law has shifted the distribution of the age of initiation of cigarette use by preventing lawful access to cigarettes.”**  We added the implication that there are some states that had state-wide Tobacco 21 laws prior to implementation of the federal law (509-510).  **“However, it should be noted that 19 states and Washington D.C. had Tobacco 21 laws that were implemented prior to the federal law”.**  We have modified our previous discussion section and this sentence to include culturally-relevant and population-based interventions (line 513).  **“...culturally-relevant interventions, and population-based interventions”** |
| 14. Page 19. The limitations section could be more robustly framed. In addition to the fact that age of initiation had to be derived using a proxy approach, there are also important implications specifically related to the longitudinal nature of the data, including loss to attrition over time and a relatively small sample that prevented more nuanced precision around certain assessed variables (e.g. race/ethnicity). It’s also important to note the age of the data. These data are from nearly a half decade ago, and there’s been considerable volatility in the tobacco product marketplace in recent years, including increasing e-cigarette use among young adults and enhanced adoption of various population-level interventions at the national/state/local levels – all of which could impact the extent of these observed findings from 2013-2016. | We appreciate the reviewer pointing out these limitations.  We agree with the reviewer that measuring age of initiation with a proxy variable is a limitation. However, due to protecting participant confidentiality, participant birth dates are not included in the PATH restricted-data. We have included this limitation in the updated manuscript in lines 520-524.  **“PATH participant birth dates are not included in the restricted-use data due to participant confidentiality, which prevented us from obtaining participants’ exact age at each wave, and is a limitation. However, we overcome this limitation by using the number of weeks between survey waves and interval-censoring to estimate the age of initiation.”**  PATH follows 32,320 adults from 2013-2014 onward. Although we limited this manuscript to 5,523 young adults never cigarette users at the first wave of PATH participation, and we respectfully disagree that this is a small sample size for a national longitudinal study. We also included 18-year-old never cigarette users who enter the study at wave 2 and 3 who had participated in the PATH youth study. Please see 108-136 and Figure 1 for inclusion criteria. In our survival analysis, we do not lose participants due to attrition because each participant contributes to the survival analysis for the amount of time that they are in the study. We understand that attrition occurs in longitudinal studies when continuous or categorical outcomes are of interest, but this is not the case with time to event analyses as in our manuscript.  PATH oversamples African Americans and Asian young adults to account for challenges in the precision of estimates by race/ethnicity. We provide the cumulative incidence of cigarette initiation by race/ethnicity with confidence intervals for each age which will allow our readers to assess the precision of the estimate at each individual age and race/ethnicity. We agree with the reviewer that we would love to have a larger sample size by each race/ethnicity and age, but this a limitation with secondary analyses. We have included this as a limitation.  **“An additional limitation is that the precision of our estimates are diminished when estimates are provided by race/ethnicity for each age due to reduced sample size, but the confidence intervals are provided to allow readers to account for the variability of the estimates.”**  We respectfully disagree that our data are from half a decade ago, as our follow-up time included 2017, and we first submitted the manuscript in 2020. PATH follows 32,320 adults from 2013-2014 onward, and for this reason it takes a year for data collection for the entire sample of participants. The inclusion criteria for our study is on lines 108-136, which is a representative sample of young adult who enter the study as never cigarette users in 2013-2016. In addition, PATH wave 4 (2016-2017) restricted-use data was not released until 2019. Therefore, we think we are attempting to disseminate our data in a timely manner.  We have modified our study to examine the impact of previous e-cigarette use on cigarette initiation based on comments from both reviewers, so we no longer consider this a limitation of our study.  While the PATH study is nationally representative, it cannot be analyzed at the state level. Therefore, we do not want to speculate how state/local interventions would be effective in our nationally representative sample given the variability in interventions across the U.S. PATH does not measure state and local interventions. We respectfully disagree that this manuscript needs to evaluate if age of initiation changes after interventions, which is beyond the scope of our study. |
| 15. It’s recommend that the authors add a clear conclusion paragraph that summarizes the major take home message for the reader and provides a clear call to action. At present, it ends with the negative aspects of the manuscript (i.e. the limitations), rather than an actionable summary and public health implications statement | We have added a conclusion paragraph to enhance the manuscript. Please see text in the manuscript and in our answer to reviewer 1 comment #10. |

References:

1.	Barrington-Trimis JL, Braymiller JL, Unger JB, et al. Trends in the Age of Cigarette Smoking Initiation Among Young Adults in the US From 2002 to 2018. JAMA Network Open. 2020;3(10):e2019022-e2019022.

2.	Perry CL, Pérez A, Bluestein M, et al. Youth or Young Adults: Which Group Is at Highest Risk for Tobacco Use Onset? The Journal of adolescent health : official publication of the Society for Adolescent Medicine. 2018;63(4):413-420.

3.	Taylor KA, Sharma E, Edwards KC, et al. Longitudinal pathways of exclusive and polytobacco cigarette use among youth, young adults and adults in the USA: findings from the PATH Study Waves 1–3 (2013–2016). Tobacco Control. 2020;29(Suppl 3):s139-s146.
