# Supplementary material for: Prospective estimation of the age of initiation of cigarettes among young adults (18–24 years old): Findings from the Population Assessment of Tobacco and Health (PATH) waves 1–4 (2013–2017)
Source: PLoS One. 2021 May 5;16(5):e0251246. doi: 10.1371/journal.pone.0251246 (PMC8099124; doi:10.1371/journal.pone.0251246)
Supplement: Attachment — Submitted filename: Rebuttal_04202021.docx [file pone.0251246.s003.docx]

**Prospective estimation of the age of initiation of cigarettes among young adults (18-24 years old): findings from the Population Assessment of Tobacco and Health (PATH) waves 1-4 (2013-2017)**

We greatly appreciate the previous feedback from the editor and reviewers, which gave us the opportunity to improve the manuscript and make it stronger.

**Journal Requirements**

**Please review your reference list to ensure that it is complete and correct. If you have cited papers that have been retracted, please include the rationale for doing so in the manuscript text, or remove these references and replace them with relevant current references. Any changes to the reference list should be mentioned in the rebuttal letter that accompanies your revised manuscript. If you need to cite a retracted article, indicate the article’s retracted status in the References list and also include a citation and full reference for the retraction notice.**
Response: We have reviewed all references and found that no papers have been retracted. We have reviewed all references and found no mistakes that need to be corrected.

**Reviewer 1**

**Comment 1. Given the events of the past year and enhanced focused on anti-racism, which the authors are clearly quite knowledgeable about given the excellent new text on this issue in the Discussion section, the manuscript text referring to racial/ethnic groups most often uses a dehumanizing approach that probably would not be corrected by a technical editor. This is when language refers solely to "Hispanics" or "Non-Hispanic Blacks" or "Non-Hispanic Others or "Non-Hispanic Whites." Preferred language occurs elsewhere when the authors refer to "Hispanic young adults" etc. because this language acknowledges the humanity of the individuals in these groups. Other strategies include terms such as "Hispanic Americans" or "Hispanic people" etc. Consistent use of this more humanizing terminology is essential.**
Response: We agree 100% with the sentiment behind this feedback. We initially left out the words “young adults” because of trying to save on word count, and we appreciate the opportunity to refer to our participants in a humanistic way. Throughout the abstract and entire manuscript, we have updated our text to refer to our participants as “[race] young adults”.

**Comment 2. The Discussion section is now excellent. The authors demonstrate superb insight in text that addresses the racial/ethnic differences in initiation in childhood and adolescent compared with the results of the present study of young adults. However, given the potential confusion that may arise from the text elsewhere in the discussion section particularly before the key text referred to in the prior sentence, it is critical to avoid confusion. For example, the sentence starting on line 374 will be more precise to say something like "...more likely to initiate ever cigarette use at an earlier age OF YOUNG ADULTHOOD than females..." The main point is in these key sentences in the Discussion to clearly specify the age range studied so these statements are not taken out of context by the cursory reader to mean overall ages of initiation rather than age of initiation in young adulthood.**
Response: We appreciate this piece of feedback and have made the change in this instance, as well as several other places in the discussion.

**Comment 3. Discussion section, line 443 "This finding is concerning given the increased health risk associated with using more than 1 tobacco product." This sentence as is should be taken out. The fact is that if the one tobacco product used is combustible tobacco cigarettes, that is the maximal risk. If a tobacco user smokes combustible tobacco cigarettes and e-cigarettes, and the e-cigarettes result in fewer combustible tobacco cigarettes this could result in reduced risk. So this is complicated and best avoided. The paragraph ends nicely with the factual statements and no additional concluding sentence is needed.**
Response: We have removed this sentence from the updated version of the manuscript.

**Comment 4. Discussion section, line 504: The authors refer to "conventional" cigarettes. This is more subjective that "combustible tobacco cigarettes" because the word "conventional" carries subjective meaning that implies "usual" or "normal". So please change to "combustible tobacco" for this reason.**
Response: We appreciate this nuance in language and as tobacco health researchers, we would never want to normalize tobacco use behaviors. We have updated this sentence to say “combustible tobacco cigarettes”.

**Reviewer #2:**

**Comment 1: The authors have adequately addressed my original comments. The only exception is original comment #14. Although I can appreciate that more recent data aren't available, which isn't particularly surprising given the glacial speed with which FDA has made data from PATH publicly available, this doesn't negate the fact that the data are old. The author are correct in stating that they aren't a half decade old - a sizable portion of it is actually more than a half decade old. Although the most recent data pull is from 2017, the baseline estimates among never smokers were pulled from data as early as 2013-2014, which was a very different time in terms of both policy and usage behaviors. The tobacco product landscape has changed considerably since 2013-2014, including since 2017. Acknowledging that these recent changes could impact more recent initiation behaviors is perfectly within the realm of possibilities and would seem like something the authors could easily insert into the limitations. For example, something like: "Respondents were followed-up as recently as 2017; however, tobacco product use behaviors have changed among youth in more recent years, including for cigarette smoking, which could have an impact more recent initiation behaviors."**
Response: We appreciate this perspective from reviewer 2 and agree that this can be considered a limitation. We have included a paraphrased version of this sentence as a limitation in the strengths and limitations section.
